# Towards simulations of long-term behavior of neural networks: Modeling synaptic plasticity of connections within and between human brain regions

**DOI:** 10.1016/j.neucom.2020.01.050

**Published:** 2020-11-27

**Authors:** Emmanouil Giannakakis, Cheol E. Han, Bernd Weber, Frances Hutchings, Marcus Kaiser

**Affiliations:** a Interdisciplinary Computing and Complex BioSystems (ICOS) research group, School of Computing, Newcastle University, Newcastle upon Tyne NE4 5TG, United Kingdom; b Institute of Neuroscience, Newcastle University, the Henry Wellcome Building, Newcastle upon Tyne NE2 4HH, United Kingdom; c Department of Electronics and Information Engineering, Korea University, Sejong, Republic of Korea; d Institute of Experimental Epileptology and Cognition Research, University of Bonn, Germany; e Department of Functional Neurosurgery, Ruijin Hospital, School of Medicine, Shanghai Jiao Tong University, Shanghai 200025, China

**Keywords:** Brain simulation, Optimization, Neural mass model, Biological neural network modeling

## Abstract

Simulations of neural networks can be used to study the direct effect of internal or external changes on brain dynamics. However, some changes are not immediate but occur on the timescale of weeks, months, or years. Examples include effects of strokes, surgical tissue removal, or traumatic brain injury but also gradual changes during brain development. Simulating network activity over a long time, even for a small number of nodes, is a computational challenge. Here, we model a coupled network of human brain regions with a modified Wilson-Cowan model representing dynamics for each region and with synaptic plasticity adjusting connection weights within and between regions. Using strategies ranging from different models for plasticity, vectorization and a different differential equation solver setup, we achieved one second runtime for one second biological time.

## Highlights

• Development of a biological neural network model that allows long term simulation of brain activity.
• Optimization of the model using multiple techniques that led to a speed-up of X200.
• Presentation of alternative simulation frameworks for long term simulations.

## 1 Introduction

The use of computer simulations in the study of the brain is becoming increasingly popular. Still, the focus of most computational studies has been to increase the number of modeled neurons and not the simulation time [1]. Thus, some recent studies [2] have managed to simulate as many as 1.73 billion neurons using supercomputers (K computer with 88,128 nodes [3]) but the biological time simulated remains very short (<1 s). For our research we followed the opposite approach using simplified brain models in order to model brain activity for as long a time as possible.

For disease processes, such as glioma growth [4], changes after stroke [5], or the emergence of epileptic seizures after a traumatic brain injury [6], effects occur over weeks, months, or years. One possibility would be to describe these events as states and transitions between states at a lower temporal resolution of only few events for each timeline. However, such an abstraction misses biological processes and interactions that can occur at shorter time-scales such as long-term plasticity. Long-term plasticity includes both synaptic plasticity (LTP and LTD) and structural plasticity. The effects of synaptic plasticity can be observed over minutes or hours [7,8] while structural plasticity [9,10] takes place over long periods of time (days or months). Thus, the computational modeling of long-term plasticity requires the development of a framework that would allow for efficient simulations of brain activity over long periods of time and with a higher temporal resolution.

Unfortunately, simulating the human brain at both high temporal and high spatial resolution, i.e. representing all neurons of the human brain, is currently impossible for longer time-scales due to the massive computational and memory requirements of such an undertaking [11]. Any model that represents single neurons, would require at least one differential equation (usually 2 or 3) to describe the dynamics of each neuron [1,12]. Additionally, given that any neuron can have tens or even hundreds of synapses whose input needs to be calculated on each computational step, the complexity and memory requirements [13] of neuronal network simulations rapidly increases as a function of the network's size and temporal resolution. The use of parallelization [14] and simplified neuron models [12], has led to significantly faster simulations but not to the extent that whole brain simulation over long time durations would require.

One way to overcome this problem, at least until computers capable of dealing with the computational and memory requirements of detailed simulations become available, is to simulate at higher temporal resolution by using the lower spatial resolution level of population models. Such models, although lacking the precision of spiking networks, which track the activity of each individual neuron, can still give a meaningful picture of whole brain activity [15], especially for longer time periods. As a proof of principle, we describe the development and implementation of a model that allowed us to simulate whole brain dynamics for 24 h of biological time, using 30 h of computational time and a temporal resolution (step-size) of 1 ms. Our study used structural connectivity between regions to develop a model of the whole brain in order to study the different effects of external stimulation on healthy and temporal lobe epileptic patients. Despite the high-level nature of our model, our simulations managed to capture differences between the two populations (the clinical details are beyond the scope of this study), suggesting that the framework we developed can be used for models of processing in healthy brains or in other species.

In addition to presenting the model we developed, we discuss various computational approaches we considered that would allow the model to simulate even longer periods of brain activity. Finally, we discuss two different options for brain simulation that could potentially be more effective and accurate than the one we used for our study.

## 2 Materials and methods

### 2.1 Structural connectivity

#### 2.1.1 Connectivity between regions

We model whole brain activity in humans for a period of 24 h by representing the brain as a network of interacting regions. Our model is based on temporal lobe epilepsy patient neuroimaging data (MRI and diffusion tensor imaging, DTI). Specifically, the neuroimaging data was used to divide the brain in 82 cortical and subcortical regions and determine the connections between them. The strength of those connections (in our model these were represented as connections between the excitatory neuron populations of the connecting regions) was initialized by the use of streamline tractography, which provided a matrix *S* of streamline counts between regions. The connectivity matrix was then initialized as:

$$W_{ij}=\left\{ \begin{array}{cc} 0.1\cdot \log\left(S_{ij}\right), & S_{ij}>0\\ 0, & S_{ij}=0 \end{array}\right.$$

where *W_ij_* the weight of the connection between regions *i* and *j*.

Moreover, the delay times between regions were calculated as the fiber trajectory length connecting two regions divided by the activity propagation speed. For the calculation of the delays we assumed the propagation of activity between regions is 7 m/s. The process of biological data collection and their manipulation is described in detail in [16]

#### 2.1.2 Connectivity and dynamics within regions

**Fig. 1:**
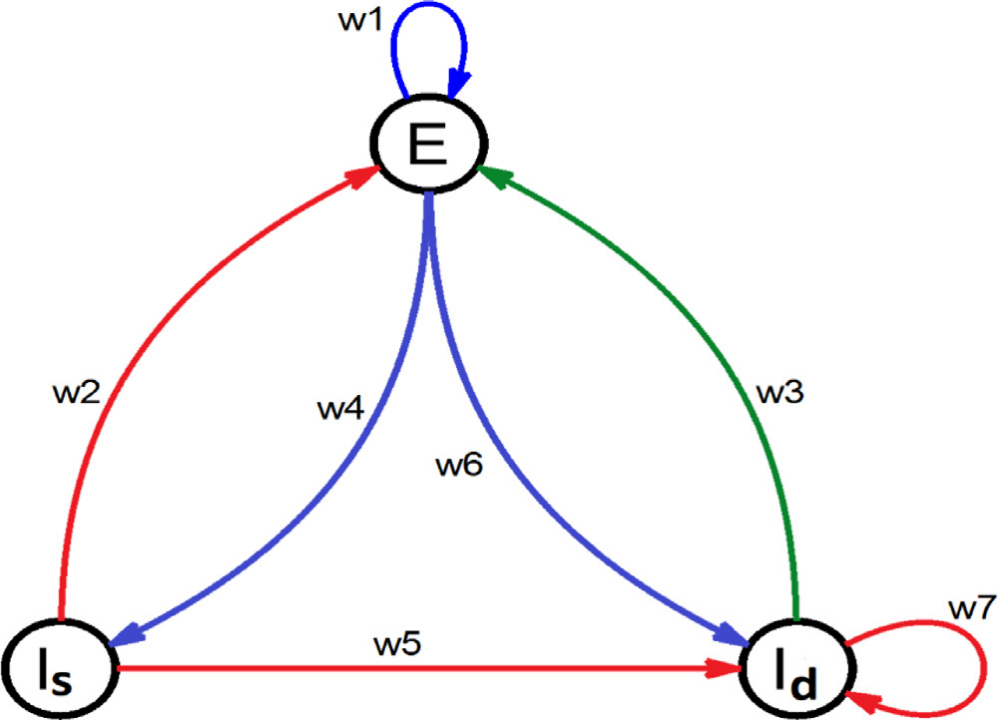
Outline of a node (brain region) in our model consisting of three neuron populations: excitatory (*E*), divisive inhibitory (*I_d_*), and subtractive inhibitory (*I_s_*). Blue arrows indicate excitatory connections while the red and green arrows indicate subtractive and divisive inhibitory connections, respectively.

**Fig. 2:**
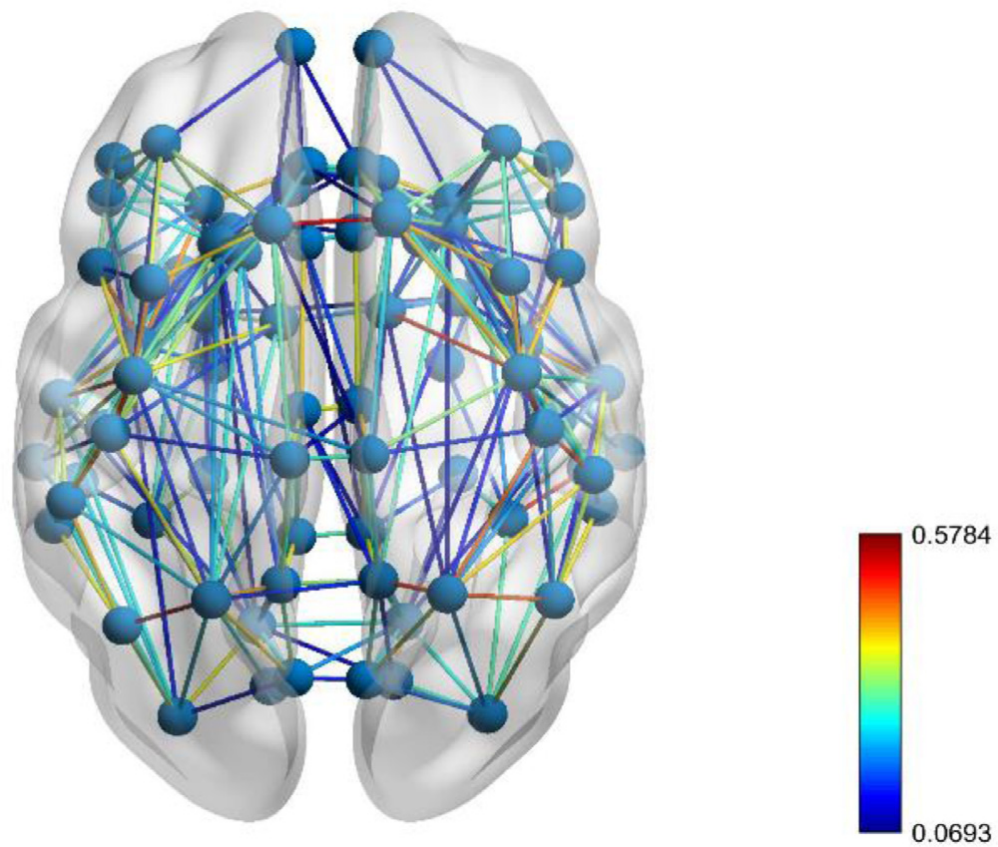
The network between brain regions that we used in our model, shown for one of the 40 subjects. The color of connections indicates the initial connection strength, as given by the logarithm of the relative number of streamlines from diffusion tensor imaging multiplied by 0.1.

The model we developed consists of 82 coupled modified Wilson–Cowan oscillators [17], each representing a brain region (Fig. 1). Each oscillator is described by the following delayed differential equations (DDE'S):

1. $\tau_{e}\frac{\partial E_{i}\left(t\right)}{\partial t}\;=\;-E_{i\;}\left(t\right)+\left(k_{e}-E_{i}\left(t\right)\right)\cdot F_{e}\left(w_{1}\cdot E_{i}\left(t\right)\;+\sum_{j=1,j\neq i}^{82}W_{ij}\cdot E_{j}\left(t-del_{ij}\right)\;+P_{e},\;w_{2}\cdot Is{\;}_{i}\left(t\right),\;w_{3}\cdot Id_{i}\left(t\right)\right)$
2. $\tau_{i}\frac{\partial Is_{i}\left(t\right)}{\partial t}\;=-Is_{i}\left(t\right)\;+\;\left(k_{i}-\;Is_{i}\left(t\right)\right)\cdot F_{i}\left(\;w_{4}\cdot E_{i}\left(t\right)+P_{s},\;0,\;0\right)$
3. $\tau_{i}\frac{\partial Id_{i}\left(t\right)}{\partial t}=\;-Id_{i}\left(t\right)\;+\;\left(k_{i}-Id_{i}\left(t\right)\right)\cdot Fi\left(w_{5}\cdot E_{i}\left(t\right)\;+\;P_{d},\;w_{6}\cdot Is_{i}\left(t\right)\;+w_{7}\cdot Id_{i}\left(t\right),\;0\right)$

where *E*_*i*_(*t*), *Is_i_*(*t*) and *Id_i_*(*t*) are the activities of the neuron populations of node *i* (excitatory, subtractive inhibitory and divisive inhibitory) at time *t, τ* is the timescale of the response of each population, *del_ij_* is the time delay between regions *i* and *j, W_ij_*, *w_k_* are the weights of the external and the internal connections respectively and *P_e_, P_s_*, *P_d_* are the external inputs of each population (Fig. 2).

The additional inhibitory population was included in order for the model to take into account the effects of divisive inhibition, which is presumed to have a gain control effect in neural populations [18]. The model we present here was developed in [19] where the effects of this additional population on the dynamics of the model are described in detail.

The input- output function *F_k_*, *k* ∈ {*e, i*} is given as a modified version of the logistic function with three-variables, representing the driver, the subtractive modulator and the divisive modulator respectively:

$$\;F_{j}\left(x,\theta ,a\right)=\frac{1}{1+\text{exp}\;\left[-\;\frac{a_{j}}{1+a}\left(x-\left(\theta_{j}+\theta \right)\right)\right]}-\frac{1}{1+\text{exp}\;\left[\frac{a_{j}\theta_{j}}{1+a}\right]}$$

Thus, in this model subtractive inhibition is represented as a displacement of the function to higher inputs and divisive modulation is represented as a decrease in the slope and maximum output of the input–output function.

The constant *k_j_*, *j* ∈ {*e, i*}, capturing the refractory dynamics of each population, is given by:

$$k_{j}=\lim_{x\to \infty }F_{j}\left(x,\theta ,a\right)=\frac{\text{exp}\;\left[\frac{a_{j}\theta_{j}}{1+a}\right]}{1+\text{exp}\;\left[\frac{a_{j}\theta_{j}}{1+a}\right]},\;j\in \left\{e,i\right\}$$

All other constants including the constants of the sigmoid functions and the external inputs are initialized according to the requirements of the simulation. The aim of our study was to examine long-term changes in brain connectivity, thus we examined several ways to implement plasticity in our model. In the different scenarios presented in the supplementary material we detail different learning rules and different combinations of internal plasticity (between the subpopulations of a region) and external plasticity (between regions) we examined during the optimization process.

### 2.2 Implementation

The aim of the simulation is: (1) to calculate solutions for the resulting system of DDE's for as long a period of time as possible; (2) to capture snapshots of the weight matrices and the activity of each node in regular intervals (in our study snapshots of activity were taken every 50 s of biological time). This is in order to examine the evolution of the system's connectivity.

After the end of the simulation, data are analyzed to determine long-term trends in the activity of selected regions as well as the overall connectivity of the brain.

In our study, the system is solved by Matlab's inbuilt dde23 delayed differential equation solver [20] using a time step of 1 millisecond. The dde23 solver uses the explicit Runge–Kutta (2,3) method (Bogacki–Shampine method, order 3 with four stages) for integration and is based on the ODE solver ode23 (single step solver). In order to run multiple simulations in parallel we used the Newcastle University Rocket HPC service (https://services.ncl.ac.uk/itservice/research/hpc/hardware/), which allowed us to simulate the brain activity of 40 subjects (each represented by a network of 82 nodes) for 24 h of biological time within a timeframe of 30 h of running time.

## 3 Results

### 3.1 Optimization

In this section we will briefly present the optimization techniques we used to speed up the simulation. A more detailed description of each simulation scenario and a detailed comparison along with pseudocode can be found in the supplementary material

In order to increase the efficiency of our code we implemented various optimization techniques and modifications to our model that lead to a significant reduction in the running time.

Our first modification that lead to a considerable reduction in the running time was to use a simplified plasticity rule that captured the essential changes induced by plasticity. Specifically, instead of representing each connection between regions by a differential equation implementing Oja's learning rule [21], we used a simplified Hebbian learning rule given as:

$$\Delta W_{ij}\left(t\right)=c\cdot E_{i}\left(t-del_{ij}\right)\cdot \left(E_{j}\left(t\right)-E_{j}\left(t-1\right)\right)$$

With subsequent normalization [8] at every update according to:

$$W_{ij}\;\leftarrow \;\frac{W_{ij}}{\sum_{i=1}^{82}W_{ij}}$$

Due to the faster speed resulting from this change, we were also able to model changes on the internal weights of the connections between populations of each node, which were updated according to a modified version of the rule we used for the external connections with subsequent normalization after every update.

$$\Delta w_{k}k^{\left(i\right)}=c\cdot Pre\left(t\right)\cdot \left(Post\left(t\right)-Post\;\left(t-1\right)\right)$$

where *Pre*(*t*), *Post*(*t*) are the activities of the presynaptic and the postsynaptic populations, respectively.

The update was initially implemented at each step and in the final versions every 10 steps, a decision that reduced the models complexity and also better represented the timescale of plasticity in actual biological networks [22].

Another important concern with the initial model was the way memory was allocated, specifically, initializing the DDE solver once and letting it perform the entire simulation was extraordinarily memory consuming since all the intermediate values where saved in memory until the end of the simulation. Since we were interested only in long term changes and thus only needed to record activity very sparsely throughout the simulation, we changed the way the DDE solver was called. Specifically, the solver is called for 10 time steps and then re-initialized with the final values the last iteration produced, given only the last *n* (here *n* = 10; n varying according to the simulation's needs) steps of the simulation as memory input using an external function. These last n-values were saved in a circular buffer architecture which overrides earlier values when new ones are added.

This step reduced the running time as well as the memory consumption of the algorithm, which, with this change, depends only on the amount of data we are recording (how often we save the values of each region). In our experiments, the recording was sparse enough to not cause concern but in the case of larger models or more frequent recording, an external disk can be used to store earlier recordings in order to save space from the working memory as in [23].

As a final optimization step, we changed the way that the input to each region is calculated by implementing a vectorized version of the initial algorithm. This final step also led to an important reduction in the running time. This version was considered effective given the available processing hardware. However, further speed-up with different hardware architectures is possible as outlined in the discussion section.

### 3.2 Complexity

**Fig. 3:**
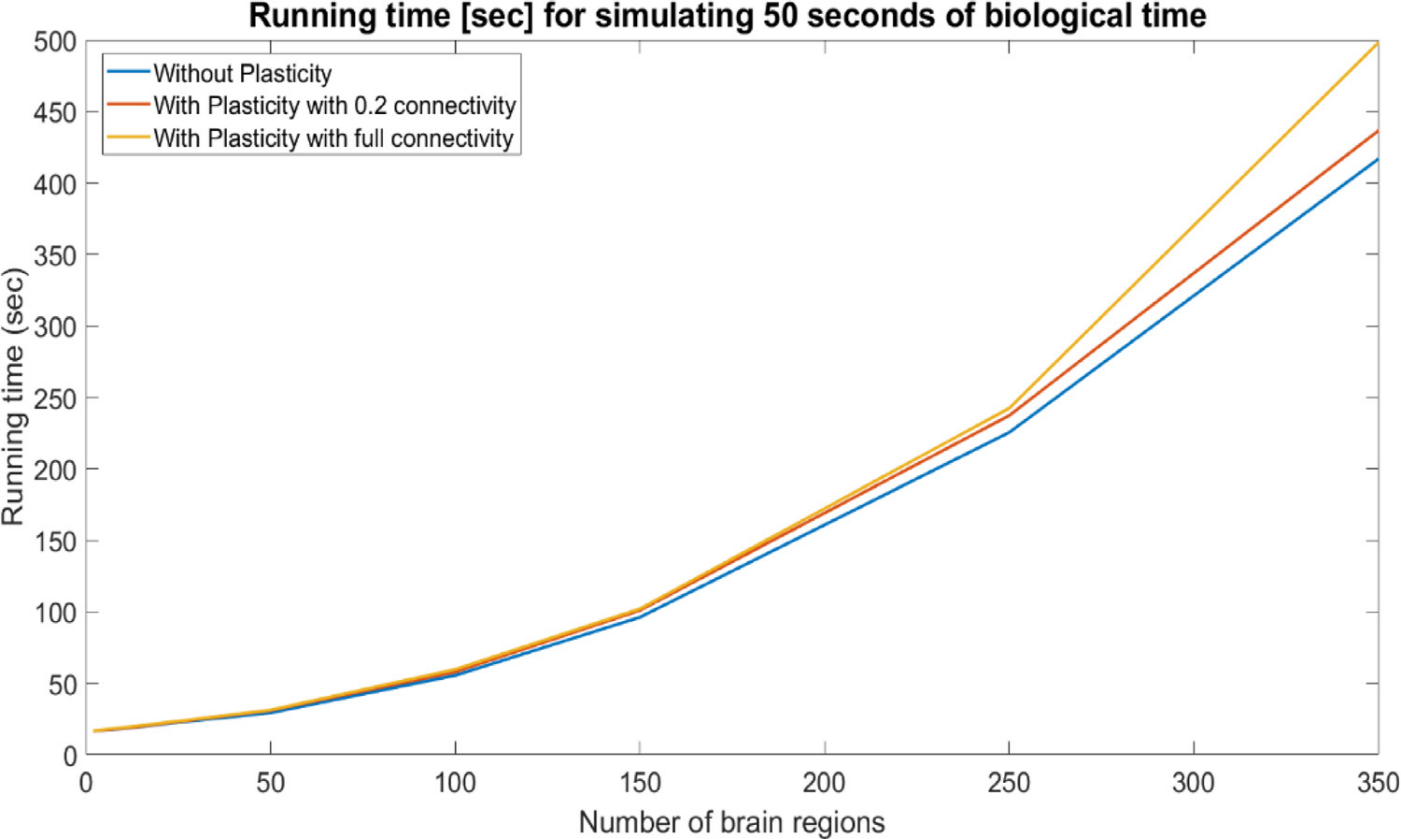
The running time required for simulating 50 s of biological time on networks of different sizes with and without plasticity for biologically realistic connectivity (*p* = 0.2) and full connectivity (*p* = 1).

To give an overview, Table 1 and Fig. 3 show the time it takes to simulate 50 s of biological time, using our final model, on networks of various sizes, with and without weight updates (internal and external).

**Table 1:** Running time [sec] for simulating 50 s of biological time under the final (and fastest) version of the model.

Number of brain regions (nodes)	Number of internal and external connections (edges)	Runtime without plasticity [seconds] (internal and external)	Runtime with plasticity [seconds] (internal and external)	Runtime with plasticity [seconds] and number of edges under full connectivity
2	14	16.76	16.80	16.80/14
10	90	18.10	18.28	18.93/150
25	300	22.64	23.03	23.45/750
50	840	29.28	30.92	31.23/2750
100	2680	55.48	57.89	59.67/10,500
150	5520	96.15	100.94	103.34/23,250
250	14,200	225.43	237.24	253.56/63,750
350	26,880	416.87	436.66	498.35/124,250

As we can see, the implementation of plasticity is not particularly time consuming, since it requires about 5% of the total running time in networks with more than 50 nodes for the original case of 0.2 connectivity (less time for smaller networks). Even in the fully connected networks (with approximately 5 times more connections that need to be updated) the required time does not increase dramatically.

The networks of different sizes are created by each region consisting of a modified Wilson-Cowan oscillator as described before (Section 2.1). In our original model, connections between regions are established with a probability of 0.2, reflecting the percentage of actual inter-region connections in the brain (about 20% of all possible connections). Here we also give the running time for a fully connected network (all possible inter-node connections are active) in order to give a more accurate picture of the model.

The overall complexity of the model is *O*(*n*^2^) (for *n* brain regions) due to the calculation of the external input for each equation (*n* regions receive input potentially from *n* regions, thus *n*^2^ computational steps).

**Fig. 4:**
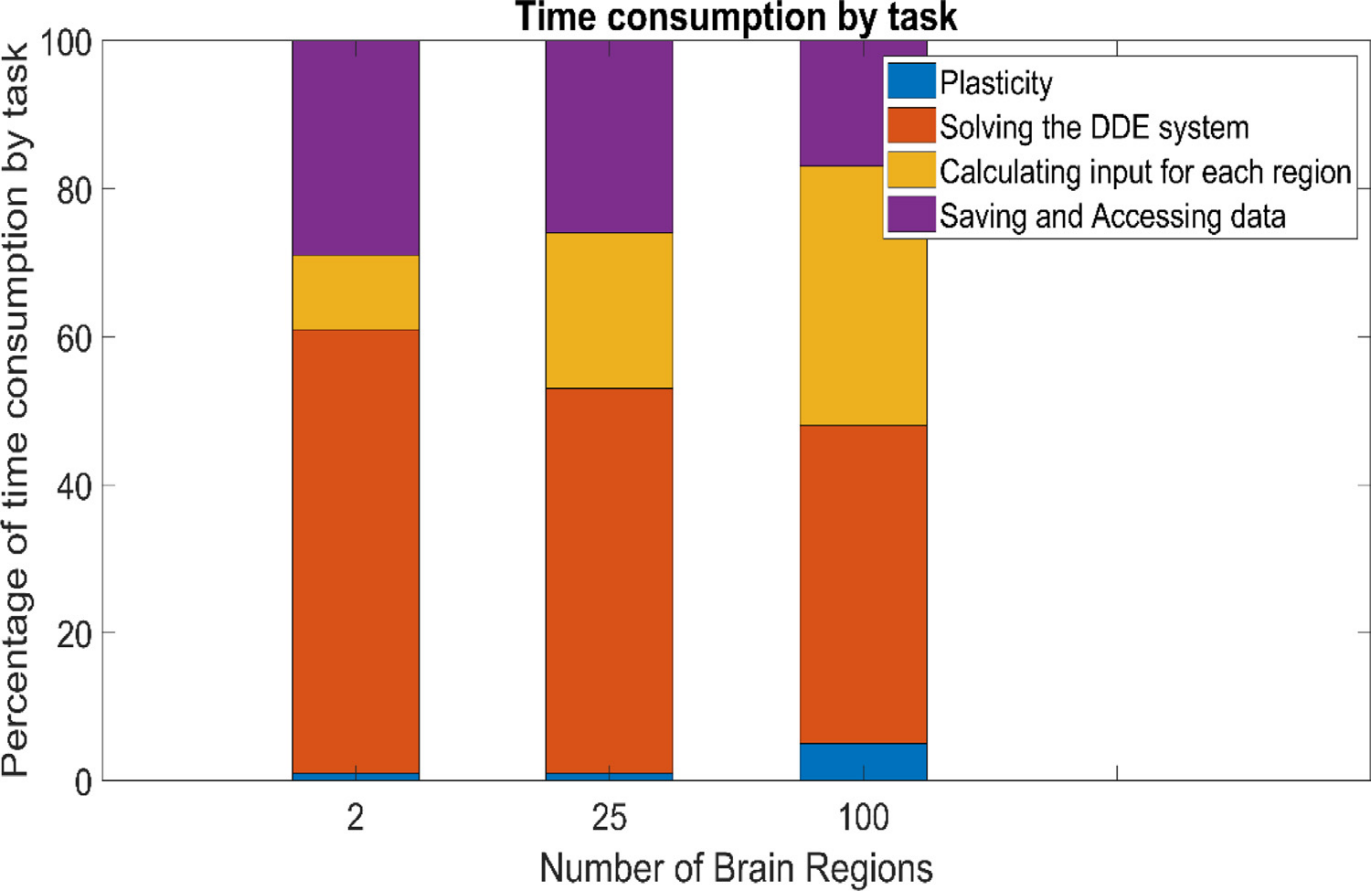
The relative proportion of algorithm runtime spent on different tasks depending on the number of brain regions. Tasks include saving and accessing data, calculating input for each region, using the delayed differential equation (DDE) solver, and updating the connection weights within and between regions (plasticity). All results are for calculations of scenario 4.

Moreover, depending on the size of the network, different factors influence the running time (Fig. 4). While in all networks the solution of the DDE system is the most time consuming task, in larger networks calculating the input for each node also requires significant amounts of time.

### 3.3 Model accuracy and limitations

Our model was used to study the long-term changes in the inter-region connectivity in healthy and epileptic subjects. Due to the lack of detailed experimental data about such changes on a brain wide scale, the parameters of the model were chosen so that the activity of each node matches the cumulative activity (average of spikes) of spiking networks simulated using the VERTEX [24] simulator. Still, despite the model being able to display realistic population dynamics, the model presented here and population models in general are appropriate only for specific tasks.

Specifically, our model cannot capture any spatial features of the activity within regions, e.g. between different cortical layers, or any aspect of the networks behavior that depend on the behavior of individual neuron types (other than the average firing rate of each population). Thus, models at this level of abstraction can only be used to study high-level aspects of network behavior (such as approximating global connectivity changes on a timescale of hours) that are dependent only on the average activity of neuronal populations.

## 4 Discussion

Our model can simulate brain dynamics and synaptic plasticity over several hours of biological time with a high temporal resolution of 1 ms. We achieved 1.26 s running time for each second of biological time through several simulation specifics: (1) using a simpler rule for weight updates, (2) not updating zero-weight connections, (3) using the DDE solver every 10 steps with the last 10 steps of data (ring memory) as input, and (4) using vectorization for repeated calculations.

Still, if we want to investigate brain activity for a period of weeks or months, the current version of the model will need to be updated in order to allow for a shorter simulation time. In this section we present several options for speeding up the implementation of our model as well as some alternatives for simulating brain activity that could potentially be more effective.

### 4.1 Model improvement: algorithm changes

The most straightforward way to reduce the implementation speed would be to increase the time step. Of course such a measure would result in reduced accuracy but given that for every snapshot of activity that is taken, 50,000 time steps of simulation are required, it may be worth considering an increased time step as a speed up option. Another possibility we considered was to use single precision floating point numbers instead of the standard double precision. This option was abandoned because the reduced accuracy could not capture the weight updates in some of the internal connections. Still, in a model with a different learning rule that does not require such great accuracy, this option is worth considering.

Another option for reducing the required amount of computations would be to use different accuracy for different nodes, i.e. to use lower accuracy for nodes representing regions whose connectivity makes significant changes unlikely. This option would be more viable in studies that focus on particular brain areas.

In addition to changing the time step, the simplification of some calculations (especially the calculation of the input from other nodes during each time step) could significantly reduce the implementation speed for larger networks. Unfortunately, all efforts to do this up to this point have been unsuccessful. Using approximation methods for some of this calculation would significantly reduce the running time but the cost would be a great loss of accuracy.

Other than making modifications in the model, some other options could be considered that could result in a much faster simulation. Given that most of the running time is spent on solving the large system of DDE's, any computing technique that could speed up the dde23 equation solver would result in a reduction of the implementation time.

Given the objective of long-term brain simulation, we should also examine models that may perform better than the one we used. Neural mass models like the Wilson Cowan model have been widely used to give representations of the average activity of large neuronal populations. Still, the simulation of long-term brain dynamics using such models is computationally costly. For this reason, especially in cases where only one aspect of brain activity is studied (connectivity), the use of even simpler models that require less computational power should be considered. An example of such an approach would be the model described in [25] which uses a simple set of differential equations that map pre-synaptic firing rates to post-synaptic activity in order to study changes in connectivity between populations of neurons. This model, by focusing on a specific aspect of brain behavior (connectivity changes) allows for much more detailed representations of connectivity than the model we used, without increasing the amount of computations that are needed for long simulations. Similar specialized models focusing on one aspect of brain dynamics can be used to achieve both increased accuracy and better implementation time for long simulations.

### 4.2 Model improvement: multi-core computing

An option we initially considered and later abandoned was the use of GPU computing. Although Matlab has a framework for the implementation of GPU computing, the differential equation solvers do not provide GPU support. If Matlab were to provide a GPU option for running the dde23 solver, the system would be solved much more efficiently.

Another option for using GPU computing would be to implement the program in another language that does provide a GPU framework for the solution of differential equations. Specifically the C++ library Odeint [26] does exactly that. Of course the transcription of hundreds of lines of Matlab code to C++ would be a significant undertaking. Moreover, given that Odeint is not explicitly designed to solve delayed differential equations as is the dde23 solver, the system will have to be adapted to account for this lack in the Odeint system, a process which may require significant changes in the way the model works. Still, given the overall advantages of GPU computing in general and of C++ in particular when handling big data [27], this is an option worth considering.

Finally an option we also considered was using Xeon Phi processors [28]. A successful biological simulation with the use of such processors is described in [29] . Although some efforts have been made to run Matlab's libraries on Xeon Phi processors, we were unable to run the dde23 solver on this system. If such an option were available in a later Matlab version, it could be used to speed up the simulation significantly. As with the GPU option, the implementation of the model in a language that is more compatible with Xeon Phi processors (FORTRAN, C, and C++) is an option worth considering.

### 4.3 Model improvement: neuromorphic computing

A promising option for long simulations with high temporal and also spatial accuracy would be the use of neuromorphic computing. Specifically, we considered the SpiNNaker system [30,31] which can model up to a billion neurons in biological time. The unique capabilities of this system could allow us to simulate actual networks with individual neurons instead of relying on neural mass models.

For a simulation similar to the one we presented, each node in the network could be represented as a small neural network of a few hundred neurons (more neurons increasing accuracy but also running time and resources required) with a ratio of 4:1 between the excitatory and the inhibitory neurons. The difference between subtractive and divisive inhibitory neurons could be modeled by differentiating inhibitory neurons according to the site (soma or dendrites) that they deliver inhibition. In this scenario, if we were still interested only in population dynamics, the activity of each population could be studied as the average activity of each neuron group.

In that way, other than being able to run a simulation in a shorter time period (a SpiNNaker machine is about 200–300 times faster than a conventional PC (Pentium 3.2 GHz PC with 1GB RAM) according to [32]), we would also be able to investigate dynamics of the brain that cannot be modeled with neural mass models. The precision and performance of SpiNNaker simulations is described in detail in [33,34]. Following the methodology presented in those papers, we estimated that the implementation of such a network using SpiNNaker would run 20−50 times faster (for 100–250 neurons per region, with firing rates from 10 to 60 Hz) than our current neural mass model implemented in Matlab.

## 5 Conclusion

We have developed a model capable of simulating plasticity-related changes in neural connectivity, both within and between regions. In order to capture long term changes in large-scale networks we have described a number of approaches for increasing the efficiency of our model. We optimized our model in several ways, which included: (1) the development of a more efficient model for plasticity; (2) using a different setup for the dde23 solver which significantly reduced the required memory; and (3) the use of vectorization techniques. Our efforts in optimizing the model led to a 200 times speedup. This model can now, therefore, provide a computationally viable platform for modeling plasticity-related changes in the brain over significant periods of time, particularly relevant for investigating long-term disease related changes.

## CRediT authorship contribution statement

**Emmanouil Giannakakis:** Writing - original draft, Software. **Cheol E. Han:** Data curation. **Bernd Weber:** Data curation. **Frances Hutchings:** Supervision, Writing - review & editing. **Marcus Kaiser:** Supervision, Writing - review & editing, Conceptualization, Methodology.

## Declaration of Competing Interest

The authors declare that they have no known competing financial interests or personal relationships that could have appeared to influence the work reported in this paper.

## Appendix Supplementary materials

Image, application 1
