# Supplementary material for: Towards simulations of long-term behavior of neural networks: Modeling synaptic plasticity of connections within and between human brain regions
Source: Neurocomputing (Amst). 2020 Nov 27;416:38–44. doi: 10.1016/j.neucom.2020.01.050 (PMC7598092; doi:10.1016/j.neucom.2020.01.050)
Supplement: Supplementary file 1 [file mmc1.docx]

**Supplementary Material**

**Optimization details**

In this section we will present in detail the steps we took to optimize the implementation of our model. For each scenario we present the main optimization idea and the improvement in the running time. The improvement in the running time for each scenario is presented in Figure S2.

Scenario 1

In the initial implementation of the model, before any optimization techniques were used, the weight of each external connection (this version did not include internal plasticity) was represented by a differential equation derived from Oja’s rule:

$\frac{\partial W_{ij}(t)}{\partial t}=c\cdot E_{j}\left( t \right)\cdot(E_{i}\left( t-del_{ij} \right)-W_{ij}\left( t \right)\cdot E_{j}\left( t \right))$

Thus, other than the differential equations representing population activity, our system also included a large number of differential equations representing connection strength. For our study this meant that the model consisted of a system of 6970 differential equations ($3\cdot82 + 82\cdot81$). Of course most of these equations were trivial since almost 85% of possible connections are not actually materialized, thus the number of non-trivial equations of the system were significantly smaller (1034 equations in a typical subject), still, the trivial equations did consume a lot of the computational time. The solution of this system was computationally very demanding and thus the running time for simulating a single second of biological time was more than 5 minutes (323.6 seconds).

Scenario 2

To speed up the simulation, we first changed the learning rule we used. Instead of treating the weights as differential equations, we started updating them in each time step using a simpler rule:

$$\Delta W_{ij}\left( t \right)=c\cdot E_{i}\left( t-del_{ij} \right)\cdot(E_{j}\left( t \right)-E_{j}\left( t-1 \right))$$

With subsequent normalization at every update according to the rule:

$$W_{ij} \leftarrow\frac{W_{ij}}{\sum_{i=1}^{82} W_{ij}}$$

Moreover, in this version of the model we introduced internal plasticity by starting to update the weights of the connections between populations of each node using the same rule we used for the external connections. Additionally, in this and all the following scenarios only the connections with non-zero weights were updated (no trivial equation updates).

This change reduced the size of the differential equations system and led to a significant decrease in the running time: In this version one second of biological time was simulated in 64 seconds, one fifth of the time required for scenario 1 to simulate the same amount of biological time.

Scenario 3

Next, we changed the method by which dde23 is used. Instead of initializing the DDE solver only once at the beginning of the simulation, for this version of the model the DDE solver is called for 10 time steps and then re –initialized with the final values the last iteration produced, given only the last n (n varying according to the simulation’s needs) steps of the simulation as memory input using an external function.

The memory function saved the last n values produced by previous iterations of the main loop in a circular buffer (ring architecture) of length n which was updated at the end of each iteration. When more than n elements were added the older elements were overwritten. In this way, the dde23 solver, when creating the Z matrix of previous values during each iteration of the loop, only used the n last values provided by the memory function, instead of using all previous values, saving memory and computational time.

Moreover, the internal and external weights were updated at the end of each iteration of the DDE solver (every 10 steps, i.e. every 10 ms of biological time) instead of updating them after every step. Reducing the update frequency was considered a biologically legitimate decision due to the fast time step we used in our equations compared with the actual timescale of plasticity in biological networks.

The pseudocode for the main script of scenarios 2 and 3 is shown in Figure S1.


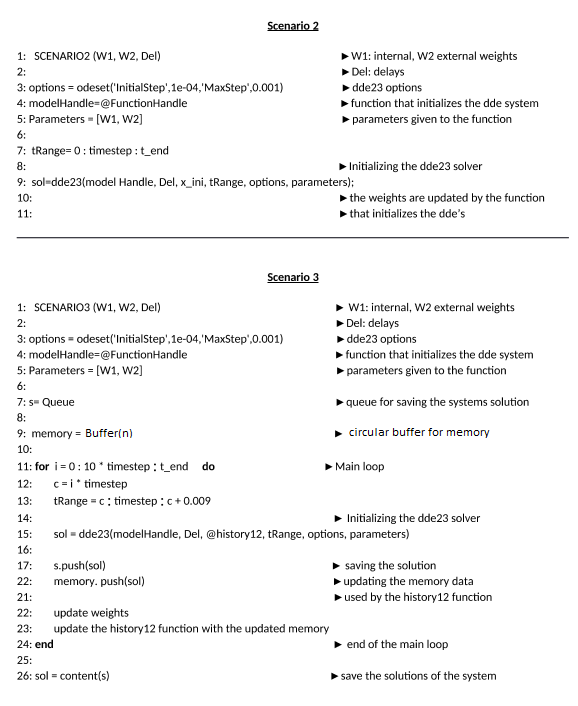


*Figure S1 – Pseudocode of the implementation of scenarios 2 and 3.*

This change reduced the running time even more. In this version one second of biological time could be simulated in 2.46 seconds of real time.


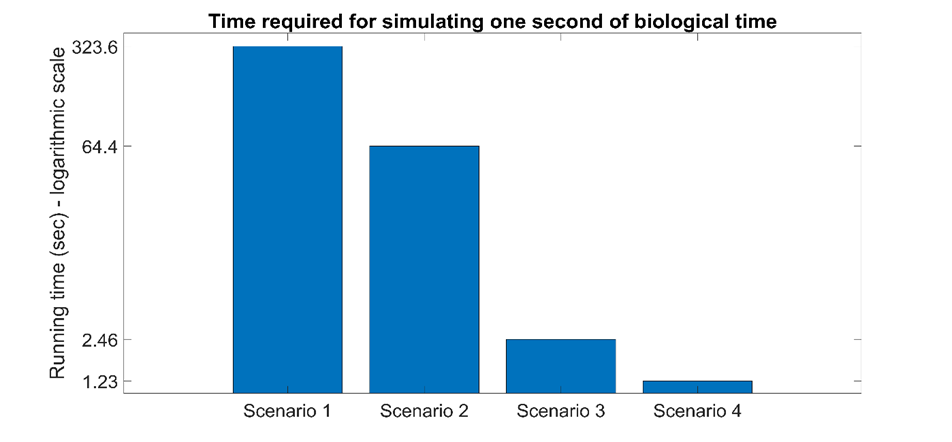


*Figure S2 – Time (in seconds) required for simulating 1 second of biological time using the different versions of our model.*

Scenario 4

For the final version of our model, we changed the way the external input in a region (from other regions) is calculated in the function that initializes the DDE system: Instead of calculating the input by using a for-loop which in each iteration adds in the total input from one region, we vectorize the outputs of each region and then calculate the sum of the resulting vector (see Figure S3).


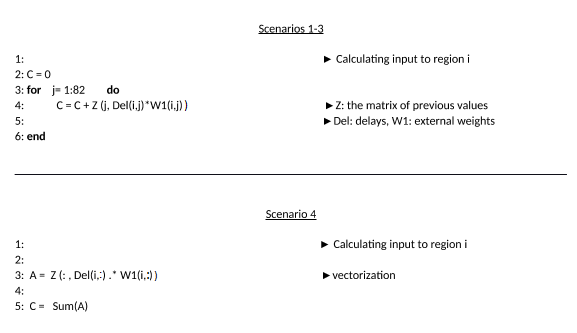


*Figure S3 – Pseudocode of the different ways of calculating the external input in each node. The vectorization implemented in scenario 4 was much more effective than the previous version*

This step reduced the running time to 1.23 seconds for simulating 1 second of biological time.

**Details of the Final Version**

In this section we will outline the parameters of the optimized model we used for our studies.

| A | Model Summary |
| --- | --- |
| Nodes | 82 (number can be modified depending on the experiment) nodes as described in (Papasavvas 2015) |
| Populations in each node | Excitatory , Divisive Inhibitory, Subtractive Inhibitory |
| Topology | Each node represents a cortical or subcortical area |
| Connectivity | Nodes are connected at the excitatory populations |
| Plasticity | Internal and External (within and between nodes) |
| Input | Constant current to each node, recurrent input from other nodes |
| Measurements | Population Activity, Connectivity changes within and between nodes |

| B | Populations | |
| --- | --- | --- |
| **Name** | **Equation** |  |
| Excitatory | $\tau_{e}\frac{\partial E_{i}\left( t \right)}{\partial t} = -E_{i}\left( t \right)+\left( k_{e}-E_{i}\left( t \right) \right)\cdot F_{e}\left( w_{1}{\cdot E}_{i}\left( t \right) +\sum_{j=1,j\neq i}^{82} W_{ij}\cdot E_{j}\left( t-del_{ij} \right) +P_{e},w_{2}\cdot Is_{i}\left( t \right), w_{3}\cdot Id_{i}\left( t \right) \right)$ |  |
| Subtractive Inhibitory | $\tau_{i}\frac{\partial Is_{i}(t)}{\partial t} =-Is_{i}\left( t \right) + \left( k_{i}- Is_{i}\left( t \right) \right)\cdot F_{i}\left( w_{4}{\cdot E}_{i}\left( t \right)+P_{s}, 0, 0 \right)$ |  |
| Divisive Inhibitory | $\tau_{i}\frac{\partial Id_{i}\left( t \right)}{\partial t}= -Id_{i}\left( t \right) + \left( k_{i}-Id_{i}\left( t \right) \right)\cdot Fi\left( w_{5}\cdot E_{i}\left( t \right) + P_{d}\boldsymbol{,}w_{6}\cdot Is_{i}\left( t \right) +w_{7}\cdot Id_{i}\left( t \right), 0 \right)$ |  |

| C | Model Parameters |
| --- | --- |
| **Name** | **Equation** |
| Sigmoid Input/Output Function | $F_{j}\left( x,\theta,a \right)=\frac{1}{1+\exp\left[ - \frac{a_{j}}{1+a}\left( x-\left( \theta_{j}+\theta\right) \right) \right]}-\frac{1}{1+\exp\left[ \frac{a_{j}\theta_{j}}{1+a} \right]}$ |
| Refractory constant | $k_{j}=\lim_{x\to\infty} F_{j}\left( x,\theta,a \right)=\frac{\exp\left[ \frac{a_{j}\theta_{j}}{1+a} \right]}{1+\exp\left[ \frac{a_{j}\theta_{j}}{1+a} \right]}, j\in\{e,i\}$ |

| D | Plasticity |
| --- | --- |
| **Name** | **Equation** |
| Between Nodes | $\Delta W_{ij}\left( t \right)=c\cdot E_{i}\left( t-del_{ij} \right)\cdot\left( E_{j}\left( t \right)-E_{j}\left( t-1 \right) \right),$ Every 10 mS |
| Within Nodes | $\Delta w_{k}k^{(i)}= c\cdot Pre\left( t \right)\cdot\left( Post\left( t \right)-Post \left( t-1 \right) \right), Every 10 mS$ |
| Normalization | $W_{a} \leftarrow\frac{W_{a}}{\sum_{i} W_{i}}$ |

| E | Measurements |
| --- | --- |
| **Activity**: The activity of each population (every node) was recorded every 50 seconds, since the main focus of the study were changes in connectivity, this was done only in order to ensure that no anomalous behavior is occurring. In studies that the focus is more on the actual behavior of each node, more frequent recording is probably needed.  **Connectivity**: Snapshots of internal and external connectivity (the weights of all connections) were saved every 50 seconds, giving a fairly detailed picture of the development of connectivity over several hours. | |

| F | Simulation Details |
| --- | --- |
| Integration method | Explicit Runge-Kutta (2,3) method |
| Step size | Each step corresponds to 1 mS of biological time. |
| Initial conditions | The activity of all nodes is set to 0 and they are activated at the beginning of the simulation by external input current. |
| Simulation time | 24 hours of biological time |
